# The efficacy of homestyle rehabilitation on negative symptoms in chronic schizophrenia: A randomized controlled trial

**DOI:** 10.3389/fpsyt.2023.1138794

**Published:** 2023-04-17

**Authors:** Jiabao Chai, Fuquan Liu, Lin Liu, Na Hu, Wenqian Huang, Hong Wang, Yonghua Cui, Hongyan Liu, Xiaojun Li, Ying Li

**Affiliations:** ^1^Beijing Huilongguan Hospital, Peking University Huilongguan Clinical Medical School, Beijing, China; ^2^Department of Psychiatry, Beijing Jishuitan Hospital, Beijing, China; ^3^Department of Psychiatry, Beijing Children’s Hospital, Capital Medical University, National Center for Children Healthy, Beijing, China; ^4^Mental Health Center of Haidian in Beijing, Beijing, China

**Keywords:** schizophrenia, negative symptoms, homestyle rehabilitation, SANS, CDSS

## Abstract

**Objective:**

Schizophrenia is a debilitating mental disorder with a high disability rate that is characterized by negative symptoms such as apathy, hyperactivity, and anhedonia that can make daily life challenging and impair social functioning. In this study, we aim to investigate the effectiveness of homestyle rehabilitation in mitigating these negative symptoms and associated factors.

**Methods:**

A randomized controlled trial was conducted to compare the efficacy of hospital rehabilitation and homestyle rehabilitation for negative symptoms in 100 individuals diagnosed with schizophrenia. The participants were divided randomly into two groups, each persisting for 3 months. The primary outcome measures were the Scale for Assessment of Negative Symptoms (SANS) and Global Assessment of Functioning (GAF). The secondary outcome measures included the Positive Symptom Assessment Scale (SAPS), Calgary Schizophrenia Depression Scale (CDSS), Simpson-Angus Scale (SAS), and Abnormal Involuntary Movement Scale (AIMS). The trial aimed to compare the effectiveness of the two rehabilitation methods.

**Results:**

Homestyle rehabilitation for negative symptoms was found to be more effective than hospital rehabilitation, according to the changes in SANS (*T* = 2.07, *p* = 0.04). Further analysis using multiple regression indicated that improvements in depressive symptoms (*T* = 6.88, *p* < 0.001) and involuntary motor symptoms (*T* = 2.75, *p* = 0.007) were associated with a reduction in negative symptoms.

**Conclusion:**

Homestyle rehabilitation may have greater potential than hospital rehabilitation in improving negative symptoms, making it an effective rehabilitation model. Further research is necessary to investigate factors such as depressive symptoms and involuntary motor symptoms, which may be associated with the improvement of negative symptoms. Additionally, more attention should be given to addressing secondary negative symptoms in rehabilitation interventions.

## 1. Introduction

Negative symptoms, which include reduced emotional expression and avolition, have been identified as core symptoms of schizophrenia (1). The severity of negative symptoms is closely linked to remission rates, life function, and quality of life (2). They play a vital role in the rehabilitation of individuals with schizophrenia (3–6). Negative symptoms can serve as essential indicators for evaluating whether a person with schizophrenia has achieved full recovery and can return to society (7). Furthermore, strong evidence suggests that negative symptoms are a significant component of schizophrenia and contribute to a considerable portion of the long-term morbidity and poor functional outcomes (2, 8, 9). Despite the use of first- and second-generation antipsychotics, their benefits on negative symptoms have been less prominent (10). Moreover, the side effects of antipsychotics, such as extrapyramidal syndrome, have come under strict scrutiny (11). As a result, psychosocial interventions for negative symptoms have attracted increasing attention (12, 13).

Rehabilitation training has been recognized as an important method for ameliorating the negative symptoms of schizophrenia, as demonstrated in various studies (14, 15). Among the existing rehabilitation programs, hospital rehabilitation, homestyle rehabilitation (which is conducted in home-like communities), and community rehabilitation have shown significant positive effects on negative symptoms. Research has indicated that community rehabilitation is more effective than hospital rehabilitation (16), and it is commonly used after symptoms resolve (17, 18). Homestyle rehabilitation is a new model that provides special apartments for 4–6 people diagnosed with schizophrenia to live together in a home-like environment with a trained caregiver assigned to their care (19, 20). While homestyle rehabilitation has been carried out in several cities in China, its efficacy in other areas, especially for negative symptoms, remains unclear. It is important to note that negative symptoms can be classified as primary or secondary. Primary negative symptoms are intrinsic to schizophrenia, while secondary negative symptoms are caused by social deprivation, depression symptoms, positive symptoms, side effects of medication, and other factors (21, 22). The efficacy of homestyle rehabilitation for factors associated with negative symptoms, such as positive symptoms and depression symptoms, also requires further investigation. Nonetheless, research suggests that homestyle rehabilitation can effectively reduce self-stigma and improve the quality of life for people diagnosed with schizophrenia (20).

The aim of this study is to examine the effectiveness of home rehabilitation as a mode of rehabilitation, specifically its impact on negative symptoms and associated factors (such as positive symptoms, depression, and extrapyramidal symptoms). Thus, the study makes three assumptions: (1) home rehabilitation is an effective mode of rehabilitation, (2) home rehabilitation produces better results in reducing negative symptoms compared to hospitalization rehabilitation, and (3) negative symptoms are influenced by several factors, among which positive symptoms, depression, and extrapyramidal symptoms are the most significant.

## 2. Methods

### 2.1. RCT design and intervention

A total of 100 inpatients with schizophrenia were recruited from September 2021 to November 2021 at the Haidian Mental Health Center, Beijing. A total of 100 patients with schizophrenia were randomly divided into the homestyle rehabilitation group and the hospital rehabilitation group, with 50 cases in each group (Create a list of all 100 participants, assigning each a unique identification number). A computer-generated random number generator was used to assign each participant to either the intervention or control group. If the random number generated is an even number, the participant is assigned to the intervention group. If the random number generated was an odd number, the participant was assigned to the control group.

**Figure 1:**
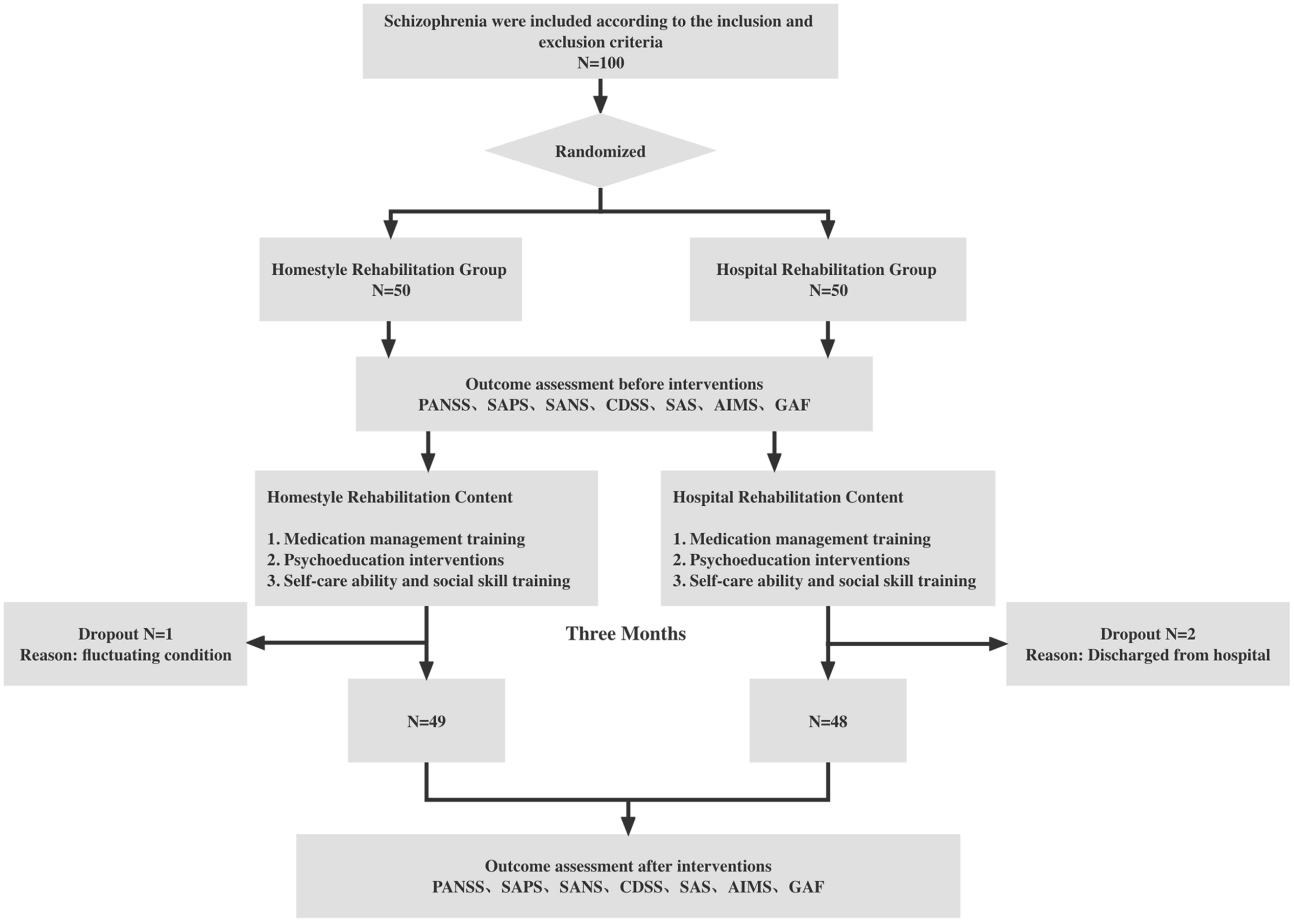
Research Roadmap.

Both groups were assessed at baseline at the time of discharge from the homestyle rehabilitation group. Subsequently, three participants dropped out (one person in the home rehabilitation group was unwilling to continue to participate, and the other two participants in the hospital rehabilitation group were unable to continue due to discharge from the hospital). Ultimately, 49 people in the homestyle rehabilitation group and 48 people in the hospital rehabilitation group completed the interventions. All inpatients included were aged between 18 and 55 and diagnosed with schizophrenia according to the ICD-10 by at least two psychiatrists. We obtained participants’ written informed consent along with their demographic and clinical information. Participants who were discharged from the hospital according to the informed consent (i.e., noncompleters) could no longer participate in the study. This study was approved by the ethics committee of the Mental Health Center of Haidian in Beijing (no. 109) (Figure 1).

**Figure 2:**
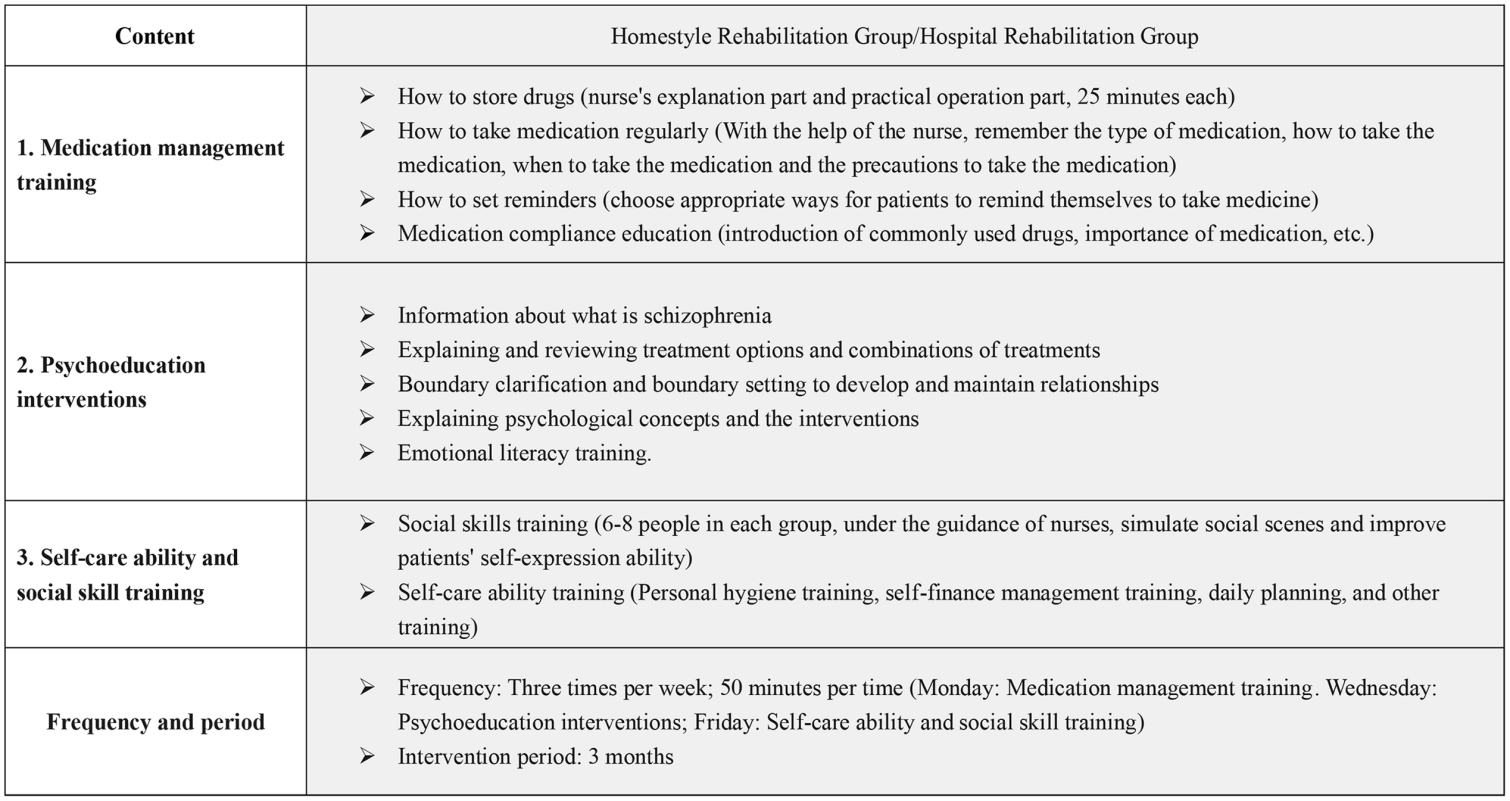
Intervention Plan Content.

Importantly, the two groups of participants underwent standardized rehabilitation training with the same contents and procedures but in different locations. The same assessment tools were used to assess all participants before the intervention and after the 3-month intervention. The two groups underwent the same standardized rehabilitation training at different locations, which mainly included (1) medication management training; (2) psychoeducation interventions; and (3) self-care ability and social skill training. The intervention period was 3 months, and the intervention frequency was three times per week. The specific content of the intervention can be found in Figure 2.

It is noteworthy that both groups of participants received standardized rehabilitation training with identical contents and procedures, albeit in separate locations. All participants were assessed using the same evaluation tools before and after the 3-month intervention. The rehabilitation program comprised three main components, namely, medication management training, psychoeducation interventions, and training in self-care ability and social skills. Both groups underwent this standardized rehabilitation training three times a week for 3 months. Please refer to Figure 2 for more details on the intervention content.

### 2.2. Inclusion criteria and exclusion criteria

Inclusion and exclusion criteria were as follows: In this study, inclusion criteria consisted of individuals who met the following criteria: (1) a diagnosis of schizophrenia according to the International Classification of Diseases, 10th edition (ICD-10); (2) stable medication for at least 2 months; (3) age between 18 and 55 years; (4) completion of junior high school or higher education; and (5) a duration of illness of at least 5 years. The exclusion criteria were as follows: (1) people with serious physical diseases; (2) people with a history of organic brain disease or head trauma, marked intellectual disability or other serious, uncontrolled physical disease; and (3) people with mental disorders caused by substance abuse such as alcohol and drugs.

### 2.3. Psychopathological assessment

All participants underwent assessment with standardized scales before and after the 3-month intervention, which were primarily evaluated by two experienced psychiatrists. The interrater agreement was determined to be good (ICC = 0.80).

#### 2.3.1. Scale for assessment of negative symptoms

The SANS (23) was utilized to evaluate the severity of negative symptoms, which include alogia, affective blunting, avolition, anhedonia, and attentional impairment. Scores range from 0 to 4, with higher scores indicating more severe negative symptoms. The SANS was used as the primary outcome measure for homestyle rehabilitation targeting negative symptoms.

#### 2.3.2. Global assessment function

The GAF scale was employed to measure the adaptive functioning of participants, with scores ranging from 0 to 100. It is commonly used to assess the effectiveness of psychiatric treatments. A higher score indicates milder disease symptoms (24). The GAF was used as the primary outcome measure for functional improvements following homestyle rehabilitation.

#### 2.3.3. Positive and negative syndrome scale

The PANSS (25) is a widely used scale that evaluates the three main dimensions of schizophrenia: positive syndrome (PANSS-P), negative syndrome (PANSS-N), and general psychopathology (GP). Scores range from 1 (“absent”) to 7 (“extreme”). The PANSS was used to assess the severity of positive symptoms, negative symptoms, and general psychopathological symptoms in enrolled participants.

#### 2.3.4. Assessment of factors associated with negative symptoms

The Assessment of Positive Symptoms (SAPS) Scale (26) assesses positive symptoms of schizophrenia and is divided into four dimensions: hallucination, delusion, positive formal thought disorder, and bizarre behavior. A 5-point Likert scale of 0–4 was used, with higher scores indicating more severe positive symptoms. The Calgary Depression Scale for Schizophrenia (CDSS) is a nine-item scale that evaluates depression independently of extrapyramidal and negative symptoms specifically for participants with schizophrenia (27, 28). The Simpson-Angus Scale (SAS) (29) is a 10-item rating scale used to measure gait (hypokinesia), rigidity, glabella tap, tremor, and salivation. The Abnormal Involuntary Movement Scale (AIMS) was also applied to assess abnormal involuntary movements, primarily in tardive dyskinesia (30). This scale consists of 12 items scored on a 5-point Likert scale ranging from 0 to 4.

### 2.4. Statistical analysis

First, we compared the baseline clinical symptoms and social function between homestyle rehabilitation and hospital rehabilitation. We assessed the normality of the data distribution using the Shapiro–Wilk test. For normally distributed data, we used *t*-tests or analysis of variance (ANOVA), and for nonnormal data, we used nonparametric analyses such as the Kruskal–Wallis test. For categorical data, we used chi-square tests. Second, we performed paired *t*-tests to compare pre- and post-intervention data between the two groups if the data were normally distributed. If the data were nonnormal, we used the Related-Samples Wilcoxon Signed Rank Test. Third, we used independent sample *t*-tests and the Kruskal–Wallis test to compare the efficacy of the two types of rehabilitation. Fourth, we conducted multiple regression analysis to identify factors that accounted for significant changes in negative symptoms. We set the significance level at 0.05.

## 3. Results

### 3.1. Comparison of demographic data before intervention

Data distribution using the Shapiro–Wilk test and the results are summarized in Supplementary Table S1. We compared the demographic data and clinical symptom-related assessments, including PANSS, SAPS, SANS, SAS, CDSS, AIMS, and GAF, of the two groups of participants at baseline (*t*-test or Kruskal–Wallis test or chi-square tests). All indexes were not significantly different. The baseline level comparison of the two groups is shown in Table 1.

**Table 1:** The basic information for homestyle rehabilitation group and hospital rehabilitation group.

	Homestyle rehabilitation group (Mean ± SD, *n* = 49)	Hospital rehabilitation group (Mean ± SD, *n* = 48)	*H*/*T*/*χ*^2^	Value of *p*
Male/Female	30/19	35/13	1.50	0.22
Age	44.90 ± 7.57	45.48 ± 9.57	0.31	0.58
Education Year	12.40 ± 3.36	11.26 ± 2.20	4.54	0.03
Duration of Illness	19.27 ± 9.03	21.56 ± 9.69	1.79	0.18
Drug dosage	263.15 ± 196.73	316.06 ± 205.23	1.30	0.20
PANSS-P	9.76 ± 2.98	9.73 ± 3.09	0.57	0.81
PANSS-N	14.67 ± 4.36	14.96 ± 5.21	0.00	0.97
PANSS-G	23.14 ± 4.33	24.17 ± 5.7	0.24	0.63
SAPS	2.33 ± 2.63	3.48 ± 5.10	0.26	0.61
SANS	19.32 ± 10.82	22.21 ± 10.34	−1.07	0.29
SAS	0.82 ± 1.39	1.29 ± 2.10	0.63	0.83
CDSS	3.24 ± 1.77	3.56 ± 1.844	0.49	0.49
AIMS	0.69 ± 0.80	0.90 ± 1.52	0.48	0.49
GAF	69.88 ± 6.09	67.88 ± 8.74	0.86	0.35

PANSS, Positive and Negative Syndrome Scale; SAPS, Scale for the Assessment of Positive Symptoms; SANS, Scale for the Assessment of Negative Symptoms; CDSS, Clinical Decision Support Systems; SAS, Self-Rating Anxiety Scale; GAF, Global Assessment of Functioning; AIMS, Abnormal Involuntary Movement Scale; SD, Standard Deviation; *H*, the statistic of Kruskal-Wallis test; *T*, the statistic of *t*-test; *χ*^2^, the statistic of chi-squared test.

### 3.2. Comparison of symptom scores before and after intervention

Before and after intervention, the results showed that participants in the homestyle rehabilitation group demonstrated significantly lower scores on the PANSS-N (*W* = −5.40, *p* < 0.001), PANSS-G (*W* = −5.90, *p* < 0.001), SANS (*T* = 8.16, *p* < 0.001), and GAF (*W* = 6.04, *p* < 0.001). A significant difference in CDSS (*W* = −5.74, *p* < 0.001) also manifested; however, no difference in other symptom scores was significant. For the hospital rehabilitation group, the results showed significant improvement on the PANSS-N (*W* = −3.91, *p* < 0.001), SAPS (*W* = −2.18, *p* = 0.03), SANS (*T* = 9.58, *p* < 0.001), and GAF (*W* = 6.00, *p* < 0.001; see Table 2 for more detailed results; *T*, the statistic of paired *t*-test; *W*, statistic of Wilcoxon signed-rank test).

**Table 2:** Pre- and post-comparison of the two groups.

Outcomes	Pro-homestyle (Mean ± SD)	Post-homestyle (Mean ± SD)	*T*/*W*	Value of *p*	Pre-hospital (Mean ± SD)	Post-hospital (Mean ± SD)	*T*/*W*	Value of *p*
PANSS-N	14.67 ± 4.36	12.12 ± 3.85	−5.40	<0.001	14.96 ± 5.21	13.71 ± 5.18	−3.91	<0.001
PANSS-P	9.76 ± 2.98	9.59 ± 2.73	−1.20	0.623	9.73 ± 3.09	9.54 ± 2.58	−0.67	0.50
PANSS-G	23.14 ± 4.36	18.86 ± 3.82	−5.90	<0.001	24.17 ± 5.70	24.00 ± 5.19	0.88	0.38
SAPS	2.33 ± 2.63	2.22 ± 2.29	0.47	0.64	3.48 ± 5.10	3.19 ± 4.74	−2.18	0.03
SANS	19.92 ± 10.82	16.80 ± 9.82	8.16	<0.001	22.21 ± 10.34	19.79 ± 9.53	9.58	<0.001
SAS	0.78 ± 1.40	0.76 ± 1.44	−0.33	0.74	1.29 ± 2.01	1.29 ± 1.97	−0.30	0.76
AIMS	0.39 ± 0.81	0.43 ± 0.91	0.45	0.66	0.96 ± 1.75	0.90 ± 1.75	−0.63	0.53
GAF	69.88 ± 6.09	75.06 ± 6.09	6.04	<0.001	67.88 ± 8.74	70.35 ± 9.48	6.00	<0.001
CDSS	3.24 ± 1.77	1.37 ± 1.06	−5.74	<0.001	3.56 ± 1.84	3.35 ± 1.63	−1.39	0.17

PANSS, Positive and Negative Syndrome Scale; SAPS, Scale for the Assessment of Positive Symptoms; SANS, Scale for the Assessment of Negative Symptoms; CDSS, Clinical Decision Support Systems; SAS, Self-Rating Anxiety Scale; AIMS, Abnormal Involuntary Movement Scale; GAF, Global Assessment of Functioning; SD, Standard Deviation; *T*, the statistic of *t*-test; *W*, statistic of Wilcoxon signed-rank test.

### 3.3. Comparison of the efficacy of the two intervention methods

By comparing the difference between the two groups of participants before and after the intervention, the results showed that in terms of PANSS-N, the homestyle rehabilitation group yielded more positive results than the hospital rehabilitation group (*H* = 11.67, *p* < 0.001), as well as the SANS (*T* = 2.07, *p =* 0.04). On the other hand, the homestyle rehabilitation group had higher scores on changes in social function than the hospital rehabilitation group based on the assessment of GAF (*H* = 10.00, *p* < 0.001; see Table 3 for details; *H*, the statistic of Kruskal-Wallis test; *T*, the statistic of *t*-test).

**Table 3:** The mean changes comparison of the two groups.

Outcomes	Changes in homestyle rehabilitation group (Mean ± SD)	Changes in hospital rehabilitation group (Mean ± SD)	*T*/*H*	Value of *p*
PANSS-N	2.55 ± 2.07	1.25 ± 2.02	11.67	<0.001
PANSS-P	0.16 ± 2.43	0.19 ± 1.82	0.18	0.90
PANSS-G	4.29 ± 3.54	0.17 ± 2.90	38.00	<0.001
SAPS	−0.04 ± 0.61	0.19 ± 0.57	3.50	0.06
SANS	3.12 ± 1.85	2.42 ± 1.45	2.07	0.04
SAS	0.02 ± 0.43	0.02 ± 0.48	0.00	1.00
AIMS	−0.02 ± 0.32	0.04 ± 0.32	0.61	0.44
GAF	5.18 ± 3.20	3.29 ± 2.16	10.00	<0.001
CDSS	1.88 ± 1.05	0.21 ± 1.00	39.45	<0.001

PANSS, Positive and Negative Syndrome Scale; SAPS, Scale for the Assessment of Positive Symptoms; SANS, Scale for the Assessment of Negative Symptoms; CDSS, Clinical Decision Support Systems; SAS, Self-Rating Anxiety Scale; AIMS, Abnormal Involuntary Movement Scale; GAF, Global Assessment of Functioning; SD, Standard Deviation; *H*, the statistic of Kruskal-Wallis test; *T*, the statistic of *t*-test.

### 3.4. Multiple regression analysis of factors associated with negative symptoms

Changes in CDSS, SAPS, SAS, and AIMS effect sizes between the pre- and postintervention were adopted as independent variables, and negative symptoms of pre- and postintervention differences were adopted as dependent variables (based on SANS). The results show that both depressive symptoms (*T* = 6.88, *p* < 0.001) and involuntary motor symptoms (*T* = 2.75, *p* = 0.007) contributed significantly to the improvement of negative symptoms (see Table 4).

**Table 4:** The regression analysis of changes of CDSS, SAS, SAPS and AIMS for the SANS.

Dependent variable (*n* = 97)	Predictors	Beta	*T*	Value of *p*
SANS (*F* = 14.16) (*p* < 0.001)	CDSS	0.58	6.88	<0.001
	SAPS	−0.04	−0.46	0.64
	SAS	0.09	1.05	0.30
	AIMS	0.23	2.75	0.007
	Constant	N/A	6.35	<0.001

CDSS, Clinical Decision Support Systems; SAPS, Scale for the Assessment of Positive Symptoms; SAS, Self-Rating Anxiety Scale; AIMS, Abnormal Involuntary Movement Scale; SANS, Scale for the Assessment of Negative Symptoms.

## 4. Discussion

In this study, both homestyle rehabilitation and hospital rehabilitation demonstrated notable efficacy in improving negative symptoms and social function in participants with schizophrenia. The between-group comparison revealed that homestyle rehabilitation was more effective than hospital rehabilitation in improving negative symptoms. Regression analysis showed that changes in depressive symptoms and improvement in involuntary motor symptoms significantly contributed to the overall efficacy.

The study aimed to investigate the effects of a multiple intervention approach consisting of drug management training, disease health education, and social skills training on the rehabilitation of individuals with chronic mental illnesses. Prior research has demonstrated that effective medication management is critical for improving compliance and reducing the risk of relapse (31, 32). Health education on diseases can also play a crucial role in improving individuals’ understanding of their conditions, particularly in addressing misunderstandings and confusion (33, 34), which can help improve negative symptoms and support disease rehabilitation (35, 36). Additionally, social skills training has emerged as a promising rehabilitation technique, with evidence suggesting that it can improve social skills, increase social support, enhance self-esteem, and reduce self-stigma, ultimately leading to the improvement of negative symptoms (37–39). Recent studies have suggested that a combination of multiple intervention methods may be more effective (40–42), and thus, our study employed a combination of all three intervention methods. The results indicated a significant improvement in negative symptoms, depression, and involuntary movement. Therefore, we recommend the wider use of multiple intervention methods in clinical practice.

In this study, we found that homestyle rehabilitation was more effective than hospital rehabilitation, and there are several possible reasons for this. First, we suspect that a change in living environment may have played a major role. Homestyle rehabilitation offers a friendly and supportive social environment, providing more opportunities for individuals to engage with the wider community. This “hospital at home” approach also enables patients to receive therapeutic interventions from hospital staff, nurses, and therapists while staying in their own homes, which is consistent with the findings of Cutleer et al. and Yanos et al., who emphasized the importance of immediate circumstances in recovery (43, 44). Furthermore, Freeman and colleagues have noted that living in an intimate social group has a positive impact on health (45). Second, we believe that reduced self-stigma and increased self-esteem resulting from living in a homestyle environment may also have contributed to the improved outcomes. Research has shown that self-stigma and self-esteem can significantly impact functional recovery (46, 47). with intrinsic stigma having a greater effect than social stigma (48). Leaving a community setting can greatly reduce the stigma associated with schizophrenia (49, 50). and a recent study by Stefano et al. (2022) found that self-stigma is closely related to subjective well-being (51). Homestyle rehabilitation also provides opportunities for patients to return to society and receive social support, which can enhance their resilience to stress by helping them reframe and cope with challenging situations (52, 53). Finally, low social functioning, limited social relationships, and poor quality of life are significant challenges faced by people with schizophrenia (54, 55), all of which may be addressed by the homestyle rehabilitation approach. These findings support the use of homestyle rehabilitation as a valuable alternative to hospital-based rehabilitation for individuals with schizophrenia.

Since both groups received the same intervention in our study, we support the statement that the homestyle rehabilitation group showed greater improvement in negative symptoms than the hospital rehabilitation group. Our results suggest that the environment may play a crucial role in improving negative symptoms, as previous studies have indicated that environmental deprivation can have an impact on negative symptoms and social networks in individuals with schizophrenia. (56–59). Our findings indicate that social environment characteristics may be strongly correlated with negative symptoms, particularly secondary negative symptoms. For instance, the poor quality of life and singular social environment in the hospitalized ward may be detrimental to patients (56). Additionally, studies have shown that hospitalized patients suffer from lower self-esteem, self-stigma, and low self-efficacy (60, 61). Therefore, efforts to make the rehabilitation environment more homestyle may be crucial for patients’ recovery from negative symptoms. In future research, the relationship between patients’ immediate environment and negative symptoms warrants attention.

Negative symptoms are a crucial factor affecting both the clinical and social prognosis of schizophrenia (9, 62). The long-term prognosis of schizophrenia depends on how well negative symptoms are controlled (9, 63). Notably, secondary negative symptoms can be influenced by positive symptoms, the environment, depressive symptoms, and motor symptoms, such as extrapyramidal or involuntary motor symptoms (22). This study found that changes in negative symptoms were associated with improvements in depressive symptoms and involuntary motor symptoms. Specifically, depressive symptoms may have a greater impact on secondary negative symptoms among negative symptoms. Previous studies have shown that improvements in motor symptoms can also improve negative symptoms (64, 65). Therefore, one possible reason for the improvement in negative symptoms may be related to secondary negative symptoms associated with depressive symptoms and involuntary motor symptoms. Based on the results of previous and current studies, we suggest that secondary negative symptoms in individuals with chronic schizophrenia should receive more attention. Furthermore, we should also pay attention to the factors that might lead to negative symptoms, especially depressive symptoms and involuntary motor symptoms.

Our study has two limitations. First, the participants were mainly from one hospital, which could limit the generalizability of our results to some extent. Second, we did not conduct any follow-up evaluations, which prevents us from assessing the long-term effect of the intervention. Moreover, data on recurrence and other indicators were not collected during participant evaluation, which should be included in future studies to fully evaluate the intervention’s effectiveness.

## 5. Conclusion

In conclusion, the homestyle rehabilitation model was more effective than the hospital rehabilitation model in improving negative symptoms in patients. The efficacy of rehabilitation for negative symptoms may be related to factors such as depressive symptoms and extrapyramidal symptoms. Future research should further examine factors, such as depressive symptoms and involuntary motor symptoms, that may be associated with the improvement of negative symptoms.

In addition, secondary negative symptoms should receive more attention in rehabilitation interventions.

## Data availability statement

The raw data supporting the conclusions of this article will be made available by the authors, without undue reservation.

## Ethics statement

The studies involving human participants were reviewed and approved by the Ethics Committees of Mental Health Center of Haidian (Beijing, no. 109). The patients/participants provided their written informed consent to participate in this study.

## Author Contributions

JC, FL, NH, WH, HL, LL, and HW were involved in the interpretation of results and manuscript preparation. XL and YL were involved in the conceptualization and design of the study. JC and FL contributed equally to this article. All authors contributed to the article and approved the submitted version.

## Funding

This work was supported by the National Natural Science Foundation of China (NSFC) under grant nos. 82001445 and 82171538, the Natural Science Foundation of Beijing Municipality under grant no. 7212035, and Beijing Hospitals Authority Youth Programme grant no. QML20211203.

## Conflict of interest

The authors declare that the research was conducted in the absence of any commercial or financial relationships that could be construed as a potential conflict of interest.

## Publisher’s note

All claims expressed in this article are solely those of the authors and do not necessarily represent those of their affiliated organizations, or those of the publisher, the editors and the reviewers. Any product that may be evaluated in this article, or claim that may be made by its manufacturer, is not guaranteed or endorsed by the publisher.
