# Supplementary material for: The efficacy of homestyle rehabilitation on negative symptoms in chronic schizophrenia: A randomized controlled trial
Source: Front Psychiatry. 2023 Apr 17;14:1138794. doi: 10.3389/fpsyt.2023.1138794 (PMC10149672; doi:10.3389/fpsyt.2023.1138794)
Supplement: Supplementary file 1 [file Table_1.docx]

**Table S1. Data distribution analysis using the Shapiro-Wilk test**

|  | Statistic | df | Sig. |
| --- | --- | --- | --- |
| Age | 0.92 | 97 | 0.00 |
| Education Year | 0.96 | 97 | 0.00 |
| Duration of Illness | 0.99 | 97 | 0.48 |
| Drug dosage | 0.98 | 97 | 0.00 |
| PANSS-P | 0.83 | 97 | 0.00 |
| PANSS-N | 0.94 | 97 | 0.00 |
| PANSS-G | 0.90 | 97 | 0.00 |
| SAPS | 0.73 | 97 | 0.00 |
| SANS | 0.98 | 97 | 0.28 |
| SAS | 0.66 | 97 | 0.00 |
| CDSS | 0.96 | 97 | 0.00 |
| AIMS | 0.66 | 97 | 0.00 |
| GAF | 0.93 | 97 | 0.00 |

*Note:* PANSS, Positive and Negative Syndrome Scale; SAPS, Scale for the Assessment of Positive Symptoms; SANS, Scale for the Assessment of Negative Symptoms; CDSS, Clinical Decision Support Systems; SAS, Self-Rating Anxiety Scale; GAF, Global Assessment of Functioning; AIMS, Abnormal Involuntary Movement Scale; SD, Standard Deviation.
